# Supplementary material for: Crustal melting in orogenic belts revealed by eclogite thermal properties
Source: Nat Commun. 2022 Aug 9;13:4673. doi: 10.1038/s41467-022-32484-w (PMC9363448; doi:10.1038/s41467-022-32484-w)
Supplement: Supplementary file 1 — Supplementary Information [file 41467_2022_32484_MOESM1_ESM.pdf]

Supplementary Information

Crustal melting in orogenic belts revealed by eclogite thermal properties

Baohua Zhang[1], Hongzhan Fei[2], Jianhua Ge[3,4], Lingsen Zeng[5], Qunke Xia[1]

List for Supplementary Figures

Supplementary Fig. 1 Geological map of the Dabie-Sulu UHP metamorphic terrane.

Supplementary Fig. 2 Microphotographs of the eclogite samples under crossed polarized light. (a) DB11, (b) DB13, (c) SL9, (d) SL12.

Supplementary Fig. 3 Effect of garnet proprotion on the thermal properties of eclogite at 1 GPa and 300–823 K.

Supplementary Fig. 4 Representative unpolarized FT-IR spectra before and after thermal property measurements. (a) garnet, (b) omphacite.

Supplementary Fig. 5 Crustal structures, composition, and radiogenic heat production in Sulu and the Himalaya-Tibet orogenic belts.

Supplementary Fig. 6 TAS (total alkali vs. silica) classification diagram for eclogites from the Dabie and Sulu terranes.

Supplementary Fig. 7 Cross section of the cell assembly for high-pressure experiments and top views of the thermocouple and impulse heater.

List for Supplementary Tables

Supplementary Table 1 Thermal diffusivity D and thermal conductivity κ of eclogites as a function of temperature and pressure.

Supplementary Table 2 Parameters derived from fitting the equations $D = D_0\,(300/T)^{nD}\,(1 + aP)$ and $\kappa = \kappa_0\,(300/T)^{nk}\,(1 + bP)$ to the experimental data in which P is in GPa and T is in K.

Supplementary Table 3 Locality, peak metamorphic condition and age, mineral assemblage, host rock, protolith age, and exhumation age for the UHP eclogites from the Dabie-Sulu terrane.

Supplementary Table 4 Major elements compositions of the UHP eclogites from the Dabie-Sulu terrane (in wt.%).

Supplementary Table 5 Chemical compositions of garnets and omphacites in the UHP eclogites from the Dabie and Sulu terrane (in wt.%).

Supplementary Table 6 Infrared spectroscopy parameters and estimated water content (in wt. ppm) of garnets and omphacites using the Beer-Lambert law.

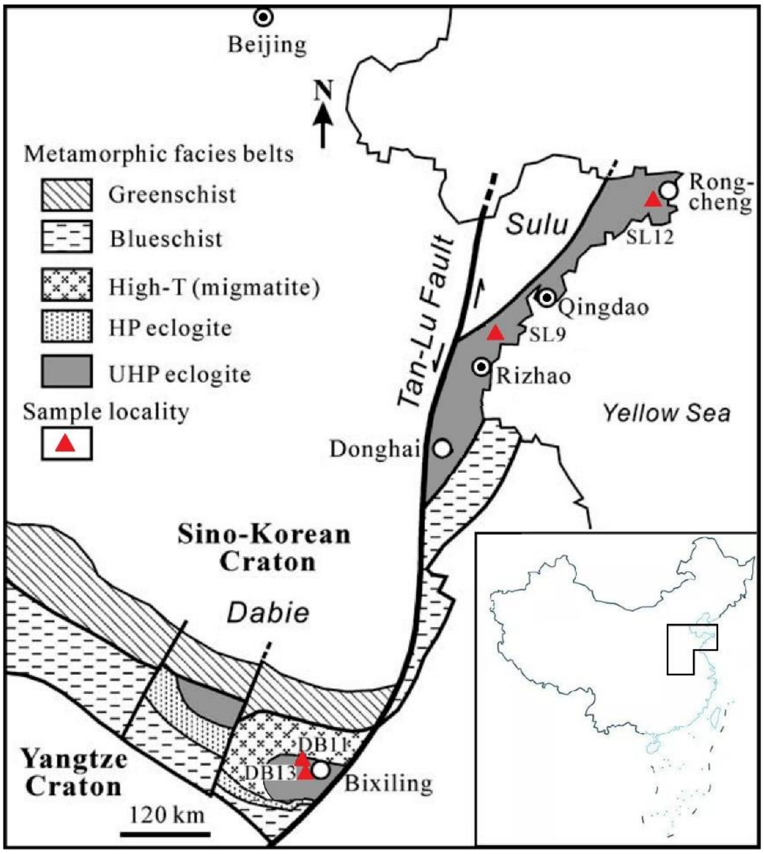

Supplementary Fig. 1 Geological map of the Dabie-Sulu UHP metamorphic terrane (Modified after Tang et al.[1]) and samples localities. Four natural eclogite samples were collected from outcrops. Reprinted from Lithos, 95, Tang, H. F., Liu, C. Q., Nakai, S. I. & Orihashi, Y, Geochemistry of eclogites from the Dabie–Sulu terrane, eastern China: new insights into protoliths and trace element behaviour during UHP metamorphism, 441-457, Copyright (2007), with permission from Elsevier.

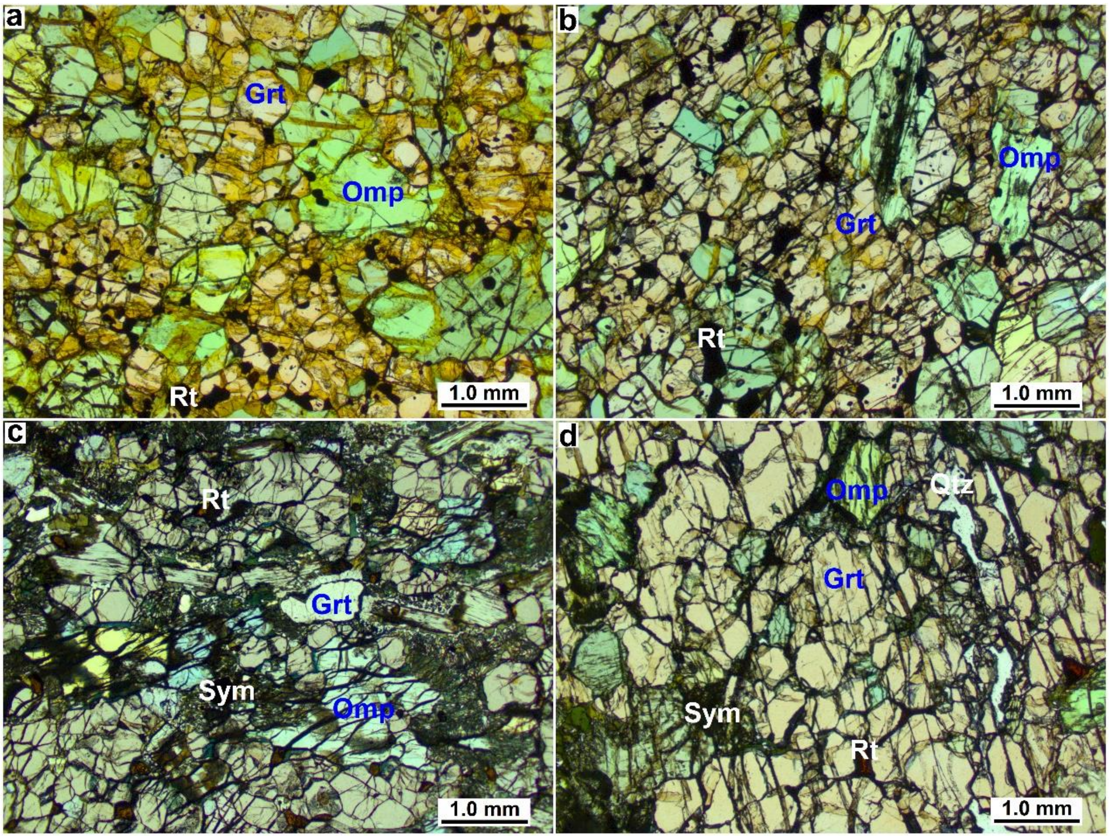

Supplementary Fig. 2 Microphotographs of the eclogite samples under crossed polarized light. (a) DB11, **(b)** DB13, **(c)** SL9, **(d)** SL12. Grt = garnet, Omp = omphacite, Rt = rutile, Qtz = quartz, Phn = phengite, Sym = symplectite, Ap = apatite, Ep = epidote, Amp = amphibole, Zo = zoisite, Ky = kyanite. Additional information is given in Supplementary Tables 3–5.

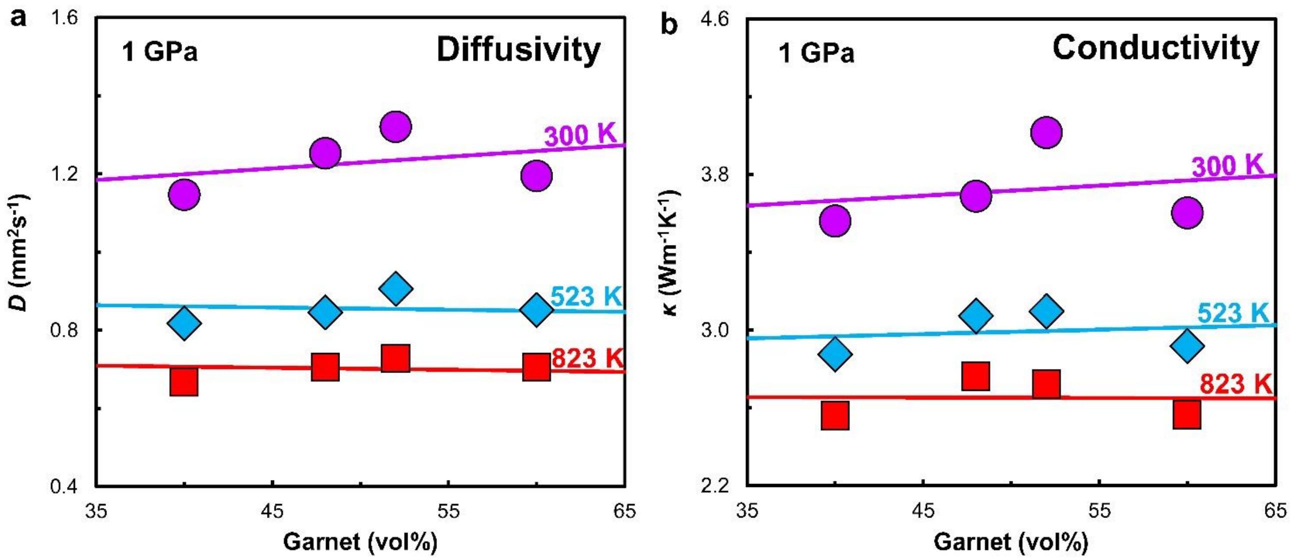

Supplementary Fig. 3 Effect of garnet proprotion on the thermal properties of eclogite at 1 GPa and 300–823 K. Effect of garnet proportion on the **(a)** thermal diffusivity and **(b)** thermal conductivity. The experimental error is less than 1.5% (Supplementary Table 1), and the error bars are smaller than the symbols.

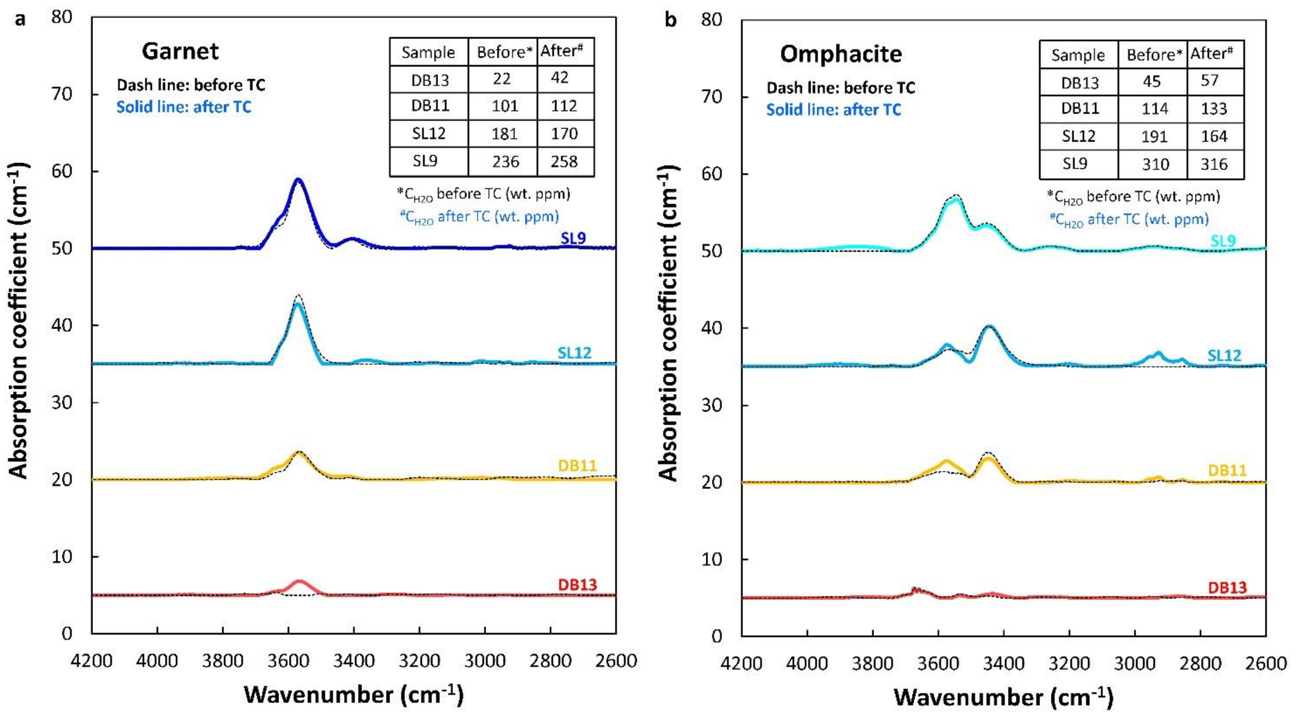

Supplementary Fig. 4 Representative unpolarized FT-IR spectra before and after thermal property measurements. (a) garnet, **(b)** omphacite. Note that, if molecular H_2 had presented, infrared absorptions at wavenumber of 4060-4100 cm^{-1} should appear[2,3], which is not the case in this study.

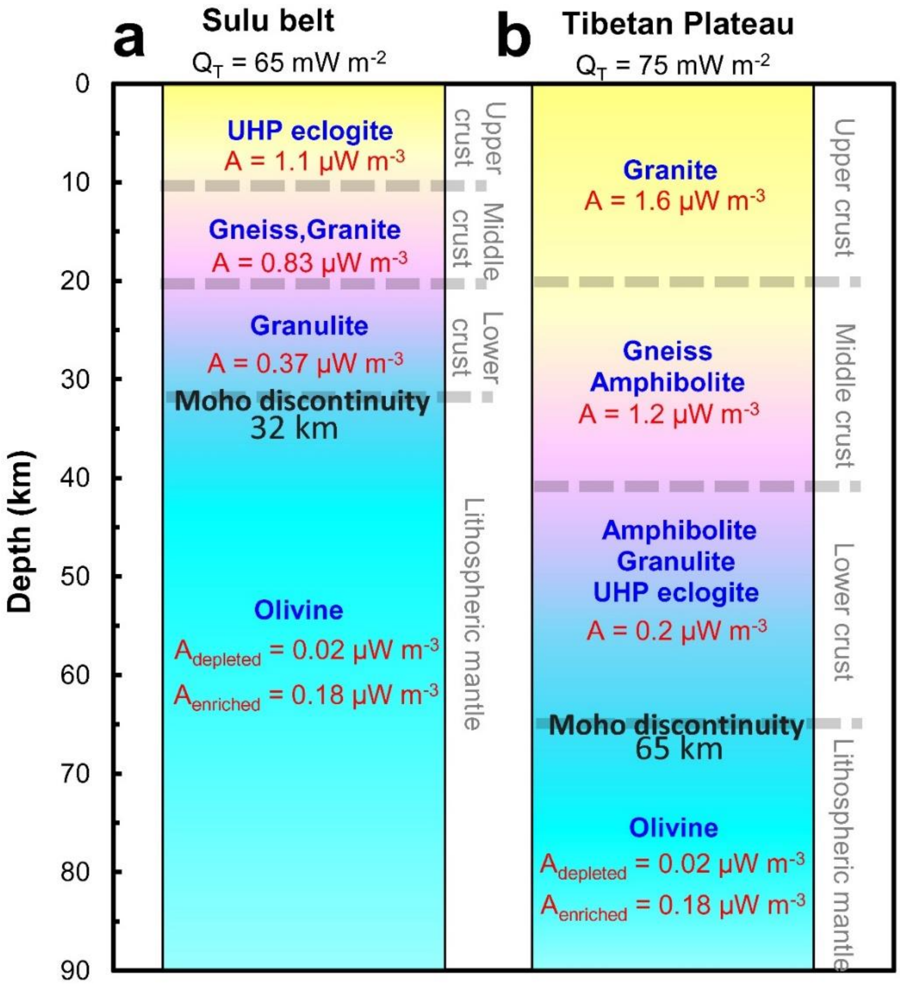

Supplementary Fig. 5 Crustal structures, composition, and radiogenic heat production in Sulu and the Himalaya-Tibet orogenic belts. All of the crusts show a three-layered structure, consisting of an upper, middle, and lower crust. The crustal structures and compositions[4–8] and average radiogenic heat production for the upper, middle and lower crust[6,9,10] were reported in previous studies. The thermal conductivity of eclogite is taken from this study and the others are previously reported data[11–14]. Depleted and enriched heat productions were applied in the lithospheric mantle[6].

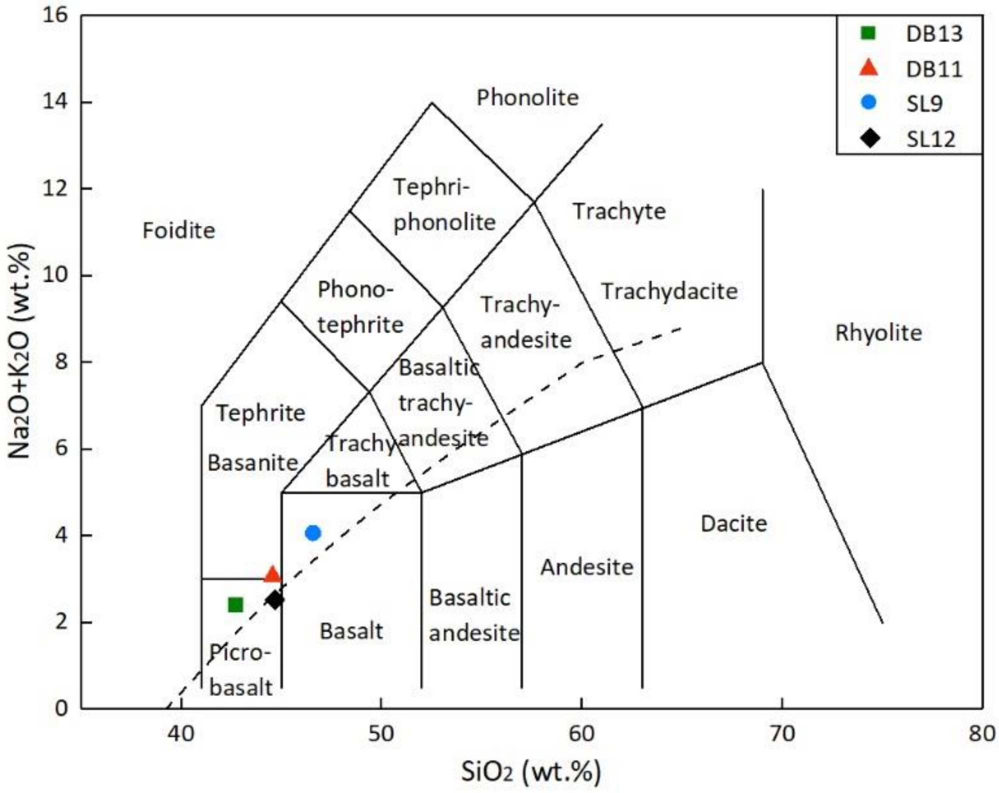

Supplementary Fig. 6 TAS (total alkali vs. silica) classification diagram for eclogites from the Dabie and Sulu terranes.

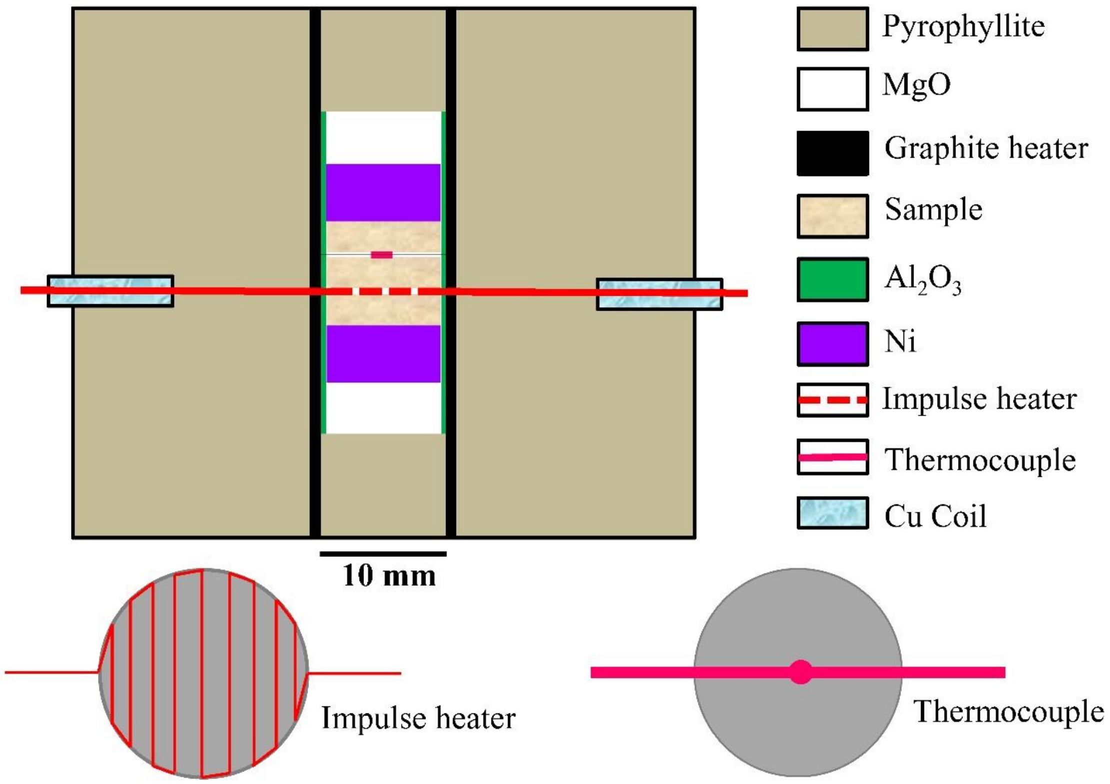

Supplementary Fig. 7 Cross section of the cell assembly for high-pressure experiments and top views of the thermocouple and impulse heater.

Supplementary Table 1 Thermal diffusivity *D* and thermal conductivity *κ* of eclogites as a function of temperature and pressure.

DB11

	1 GPa		2 GPa		3 GPa	
T (K)	*D* ($mm^2 s^{-1}$)	*κ* ($Wm^{-1} K^{-1}$)	*D* ($mm^2 s^{-1}$)	*κ* ($Wm^{-1} K^{-1}$)	*D* ($mm^2 s^{-1}$)	*κ* ($Wm^{-1} K^{-1}$)
300	1.253(6)	3.688(8)	1.423(3)	3.923(5)	1.516(4)	4.120(6)
323	1.184(6)	3.609(10)	1.278(3)	3.807(5)	1.398(4)	3.993(5)
373	1.070(5)	3.496(9)	1.147(3)	3.679(5)	1.259(3)	3.795(6)
423	0.965(6)	3.291(10)	1.068(4)	3.531(6)	1.146(4)	3.647(6)
473	0.897(7)	3.213(14)	0.988(4)	3.378(8)	1.066(5)	3.509(8)
523	0.846(9)	3.073(17)	0.943(5)	3.291(9)	0.995(5)	3.415(9)
573	0.811(11)	2.988(23)	0.885(7)	3.170(12)	0.964(9)	3.311(10)
623	0.773(11)	2.956(18)	0.839(7)	3.113(14)	0.919(8)	3.253(14)
673	0.752(10)	2.874(29)	0.825(8)	3.085(16)	0.862(8)	3.201(15)
723	0.738(11)	2.795(20)	0.788(8)	2.968(18)	0.843(10)	3.085(14)
773	0.723(9)	2.789(21)	0.766(9)	2.936(20)	0.811(9)	3.055(18)
823	0.705(11)	2.764(23)	0.754(10)	2.880(24)	0.804(10)	3.003(20)

DB13

	1 GPa		2 GPa		3 GPa	
T (K)	*D* ($mm^2 s^{-1}$)	*κ* ($Wm^{-1} K^{-1}$)	*D* ($mm^2 s^{-1}$)	*κ* ($Wm^{-1} K^{-1}$)	*D* ($mm^2 s^{-1}$)	*κ* ($Wm^{-1} K^{-1}$)
300	1.321(7)	4.016(18)	1.485(10)	4.562(31)	1.649(10)	5.111(32)
323	1.224(7)	3.706(17)	1.375(8)	4.346(24)	1.532(9)	4.939(29)
373	1.084(5)	3.495(13)	1.214(7)	4.005(21)	1.349(7)	4.500(22)
423	1.031(5)	3.325(14)	1.150(7)	3.700(19)	1.268(7)	4.195(20)
473	0.932(5)	3.189(14)	1.058(6)	3.610(18)	1.183(6)	3.932(19)
523	0.906(4)	3.097(11)	0.972(7)	3.392(19)	1.067(6)	3.789(18)
573	0.840(4)	2.966(13)	0.954(5)	3.322(15)	1.034(7)	3.695(19)
623	0.825(5)	2.882(14)	0.896(6)	3.245(17)	1.011(6)	3.545(19)
673	0.793(6)	2.856(17)	0.863(7)	3.125(20)	0.941(7)	3.446(19)
723	0.777(7)	2.771(17)	0.850(7)	3.080(23)	0.907(7)	3.401(23)
773	0.744(7)	2.865(24)	0.819(7)	3.011(23)	0.887(7)	3.336(21)
823	0.728(7)	2.722(29)	0.789(7)	2.993(29)	0.871(9)	3.296(22)

SL9

	1 GPa		2 GPa		3 GPa	
T (K)	*D* ($mm^2 s^{-1}$)	*κ* ($Wm^{-1} K^{-1}$)	*D* ($mm^2 s^{-1}$)	*κ* ($Wm^{-1} K^{-1}$)	*D* ($mm^2 s^{-1}$)	*κ* ($Wm^{-1} K^{-1}$)
300	1.147(5)	3.651(15)	1.209(8)	3.797(23)	1.268(7)	4.033(24)
323	1.102(5)	3.484(15)	1.160(5)	3.672(17)	1.225(5)	3.862(16)
373	0.985(5)	3.274(15)	1.040(5)	3.442(14)	1.097(6)	3.635(18)
423	0.900(5)	3.114(14)	0.981(6)	3.272(19)	1.019(6)	3.439(18)
473	0.842(6)	2.979(18)	0.904(7)	3.129(19)	0.954(7)	3.265(25)
523	0.798(8)	2.876(22)	0.859(9)	3.039(26)	0.916(6)	3.194(20)
573	0.758(7)	2.771(23)	0.819(7)	2.960(22)	0.864(8)	3.083(23)
623	0.716(8)	2.755(27)	0.786(9)	2.869(41)	0.812(8)	3.017(24)

673	0.713(10)	2.648(33)	0.750(9)	2.809(25)	0.791(9)	2.933(25)
723	0.680(11)	2.605(31)	0.724(10)	2.750(29)	0.759(6)	2.883(17)
773	0.671(13)	2.558(41)	0.702(9)	2.730(27)	0.734(6)	2.828(17)
823	0.667(10)	2.559(31)	0.691(7)	2.673(20)	0.722(6)	2.772(15)
873	0.655(9)	2.524(26)	0.678(8)	2.661(21)	0.700(7)	2.764(21)

SL12

	1 GPa		2 GPa		3 GPa	
T (K)	D (mm^2 s^{-1})	*κ* (Wm^{-1} K^{-1})	*D* (mm^2 s^{-1})	*κ* (Wm^{-1} K^{-1})	*D* (mm^2 s^{-1})	*κ* (Wm^{-1} K^{-1})
300	1.195(6)	3.603(24)	1.328(8)	3.855(27)	1.471(7)	4.219(26)
323	1.106(6)	3.482(19)	1.226(9)	3.709(19)	1.364(7)	4.096(24)
373	1.014(7)	3.298(16)	1.106(5)	3.572(21)	1.237(7)	3.854(26)
423	0.942(5)	3.163(20)	1.024(9)	3.389(25)	1.134(8)	3.719(30)
473	0.857(4)	3.019(21)	0.948(6)	3.247(30)	1.069(7)	3.491(37)
523	0.832(5)	2.918(18)	0.899(5)	3.119(33)	0.985(8)	3.353(23)
573	0.805(5)	2.817(17)	0.873(5)	3.060(19)	0.908(8)	3.284(22)
623	0.758(5)	2.785(21)	0.831(6)	2.971(20)	0.958(7)	3.138(25)
673	0.742(6)	2.721(20)	0.794(6)	2.888(23)	0.879(8)	3.056(23)
723	0.733(6)	2.652(23)	0.779(6)	2.785(25)	0.852(9)	3.008(26)
773	0.712(6)	2.561(23)	0.765(7)	2.769(26)	0.824(8)	2.926(24)
823	0.706(7)	2.563(35)	0.751(6)	2.668(25)	0.812(8)	2.828(25)
873	0.688(6)	2.509(27)	0.734(6)	2.663(25)	0.799(9)	2.803(25)

Supplementary Table 2 Parameters derived from fitting the equations $D = D_0 (300/T)^{nD} (1 + aP)$ and $\kappa = \kappa_0 (300/T)^{nk} (1 + bP)$ to the experimental data in which P is in GPa and T is in K.

Sample	P (GPa)	$D = D_0 (300/T)^{nD} (1 + aP)$				$\kappa = \kappa_0 (300/T)^{nk} (1 + bP)$			
		D_0 ($mm^2 s^{-1}$)	n_D	a (GPa^{-1})	R^2	κ_0 ($Wm^{-1}K^{-1}$)	n_k	b (GPa^{-1})	R^2
DB11	1	1.134	0.599	0.116	0.993	3.478	0.307	0.062	0.991
DB11	2	1.134	0.632	0.116	0.990	3.478	0.307	0.062	0.991
DB11	3	1.134	0.647	0.116	0.996	3.478	0.327	0.062	0.997
DB13	1	1.157	0.593	0.142	0.992	3.468	0.436	0.158	0.988
DB13	2	1.157	0.628	0.142	0.993	3.468	0.469	0.158	0.994
DB13	3	1.157	0.651	0.142	0.994	3.468	0.486	0.158	0.995
SL9	1	1.087	0.563	0.056	0.992	3.325	0.340	0.236	0.997
SL9	2	1.087	0.565	0.056	0.999	3.325	0.343	0.236	0.999
SL9	3	1.087	0.572	0.056	0.999	3.325	0.361	0.236	0.998
SL12	1	1.055	0.526	0.131	0.989	3.276	0.348	0.094	0.997
SL12	2	1.055	0.565	0.131	0.991	3.276	0.373	0.094	0.992
SL12	3	1.055	0.590	0.131	0.995	3.276	0.387	0.094	0.993

Supplementary Table 3 Locality, peak metamorphic condition and age, mineral assemblage, host rock, protolith age, and exhumation age for the UHP eclogites from the Dabie-Sulu terrane.

Sample	Locality	Peak metamorphic condition and age	Mineral assemblage (modal abundance)	Host rock (type*)	Protolith age	Exhumation age
DB11	Bixiling	883–973 K, >2.7 GPa[15], 225-235 Ma[16]	Grt(48), Omp(45), Rt(2), Qtz(2), Phn(2)	Ultramafic rock(III)	657–757 Ma[18]	230 Ma[15]
DB13	Bixiling	883–973 K , >2.7 GPa[15], 225-235 Ma[16]	Grt(52), Omp(40), Rt(2), Qtz(2), (Qtz, Ep, Ky)	Ultramafic rock(III)	657–757 Ma[18]	230 Ma[15]
SL9	Zhucheng	963–1093 K, 3.0 GPa[17], 225-235 Ma[16]	Grt(40), Omp(30), Sym(20), Phn(3), Ap(2), (Rt, Zo, Ky)	Gneiss(I)	788 $\pm$ 10 Ma[18]	230 Ma[15]
SL12	Rongcheng	1073 K, 3.4 GPa[17], 225-235 Ma[16]	Grt(60), Omp(25), Sym(10), Qtz(2), Amp(2), (Rt)	Ultramafic rock(III)	747 $\pm$ 13 Ma[18]	230 Ma[15]

Grt = garnet, Omp = omphacite, Rt = rutile, Qtz = quartz, Phn = phengite, Sym = symplectite, Ap = apatite, Ep = epidote, Amp = amphibole, Zo = zoisite, Ky = kyanite. Minerals in parentheses occur in small amounts, approximately 1% or less.

*Following the same classified by Tang et al.[1] and references therein.

Supplementary Table 4 Major elements compositions of the UHP eclogites from the Dabie-Sulu terrane (in wt.%).

	DB11	DB13	SL9	SL12
SiO_2	44.55	42.68	46.57	44.67
TiO_2	2.30	2.55	1.96	1.90
Al_2O_3	15.32	13.72	16.47	15.91
Fe_2O_3*	16.67	19.16	15.46	17.25
MnO	0.21	0.27	0.28	0.27
MgO	7.74	7.80	5.28	6.32
CaO	10.79	9.88	9.11	11.69
Na_2O	2.99	2.34	3.65	2.45
K_2O	0.09	0.08	0.42	0.08
P_2O_5	0.02	0.06	0.40	0.05
LOI	0.03	0.52	0.33	0.02
Total	100.71	99.06	99.93	100.61

Fe_2O_3* represents that the total iron is given as Fe_2O_3.

Supplementary Table 5 Chemical compositions of garnets and omphacites in the UHP eclogites from the Dabie and Sulu terrane (in wt.%).

Sample	DB11		DB13		SL9		SL12	
	Garnet	Omphacite	Garnet	Omphacite	Garnet	Omphacite	Garnet	Omphacite
SiO_2	38.33	53.85	38.60	53.71	38.68	57.65	38.72	52.85
TiO_2	0.06	0.10	0.02	0.01	0.03	0.06	0.10	0.09
Al_2O_3	20.30	7.52	20.34	6.13	20.12	10.50	19.68	7.84
Cr_2O_3	0.15	0.04	0.03	0.02	0.03	0.01	0.02	0.01
FeO	20.60	5.92	24.88	6.30	25.07	6.72	22.83	6.11
MnO	0.35	0.02	0.31	0.03	0.50	0.04	0.36	0.03
MgO	7.57	11.22	7.98	12.32	6.73	8.29	6.24	10.59
CaO	11.63	13.87	7.85	15.28	8.83	8.52	12.38	15.64
Na_2O	0.06	7.58	0.12	5.62	0.07	8.69	0.06	6.91
Total	99.05	100.12	100.13	99.42	100.06	100.48	100.39	100.07

Supplementary Table 6 Infrared spectroscopy parameters and estimated water content (in wt. ppm) of garnets and omphacites using the Beer-Lambert law.

Parameter	DB11		DB13		SL9		SL12	
	Garnet	Omphacite	Garnet	Omphacite	Garnet	Omphacite	Garnet	Omphacite
Δ(cm^{-2})§	1.97/2.02	3.77/4.09	0.43/0.80	1.49/1.75	4.59/4.66	10.26/9.71	3.52/2.60	6.32/4.26
I(ppm^{-1} cm^{-2})	1.39	7.09	1.39	7.09	1.39	7.09	1.39	7.09
t(cm)§	0.014/0.013	0.014/0.013	0.014/0.013	0.014/0.013	0.014/0.013	0.014/0.013	0.014/0.011	0.014/0.011
γ	1	1/3	1	1/3	1	1/3	1	1/3
C_{H2O} (wt. ppm)§	101/112	114/133	22/44	45/57	236/258	310/316	181/170	191/164
C_{H2O} (wt. ppm)※	100/113		29/44		187/198		156/143	

Note: The water content in each mineral was calculated using the Beer–Lambert law $C_{H2O} = \Delta/(I \times t \times \gamma)$[19], where C_{H2O} is the content of hydrogen species (wt. ppm H_2O), Δ is the integrated area of the -OH absorption bands (cm^{-2}), I is the integral specific absorption coefficient (ppm^{-1} cm^{-2}), t is the thickness (cm), and γ is the orientation factor.

§Values before and after the forward slash represent water contents determined before and after thermal property measurements;

※Values before and after the forward slash indicate the initial and final bulk water contents of four eclogites for each run based on mineral assemblage in Supplementary Table 3.

Supplementary References

1. Tang, H. F., Liu, C. Q., Nakai, S. I. & Orihashi, Y. Geochemistry of eclogites from the Dabie–Sulu terrane, eastern China: new insights into protoliths and trace element behaviour during UHP metamorphism. *Lithos* **95**, 441–457 (2007).
2. Yang, X., Keppler, H., & Li, Y. Molecular hydrogen in mantle minerals. *Geochem. Perspect. Lett*. **2**, 160–168 (2016).
3. Moine, B. N., et al. Molecular hydrogen in minerals as a clue to interpret ∂D variations in the mantle. *Nature Commun.* **11**, 3604 (2020).
4. Owens, T. J. & Zandt, G. Implications of crustal property variations for models of Tibetan plateau evolution. *Nature* **387**, 37–43 (1997).
5. Hacker, B. R. et al. Hot and dry deep crustal xenoliths from Tibet. *Science* **287**, 2463–2466 (2000).
6. He, L., Hu, S., Yang, W. & Wang, J. Radiogenic heat production in the lithosphere of Sulu ultrahigh-pressure metamorphic belt. *Earth Planet. Sci. Lett.* **277**, 525–538 (2009).
7. Wang, C. Y., Chen, W. P. & Wang. L. P. Temperature beneath Tibet. *Earth Planet. Sci. Lett.* **375**, 326–337 (2013).
8. Li, W. et al. Petrofabrics and seismic properties of Himalayan amphibolites: Implications for a thick anisotropic deep crust beneath southern Tibet. *J. Geophys. Res.* **125**, e2019JB018700 (2020).
9. Chi, Q. H. & Yan, M. C. Radioactive elements of rocks in North China Platform and the thermal structure and temperature distribution of the modern continental lithosphere. *Chin. J. Geophys.* **41**, 38–48 (1998).
10. Goes, S., Hasterok, D., Schutt, D. L. & Klöcking, M. Continental lithospheric temperatures: A review. *Phys. Earth Planet. Inter.* **306**, 106509 (2020).

11. Seipold, U. & Huenges, E. Thermal properties of gneisses and amphibolites–high pressure and high temperature investigations of KTB-rock samples. *Tectonophys.* **291**, 173–178 (1998).

12. Merriman, J. D., Whittington, A. G., Hofmeister, A. M., Nabelek, P. I. & Benn, K. Thermal transport properties of major Archean rock types to high temperature and implications for cratonic geotherms. *Precam. Res.* **233**, 358–372 (2013).

13. Fu, H. F. et al. Thermal diffusivity and thermal conductivity of granitoids at 283–988 K and 0.3–1.5 GPa. *Am. Mineral.* **104**, 1533–1545 (2019).

14. Zhang, B. H., Ge, J. H., Xiong, Z. L. & Zhai, S. M. Effect of water on the thermal properties of olivine with implications for lunar internal temperature. *J. Geophys. Res.* **124**, 3469–3481 (2019).

15. Zhang, R. Y., Liou, J. G. & Ernst, W. G. The Dabie–Sulu continental collision zone: a comprehensive review. *Gondwana Res.* **16**, 1–26 (2009).

16. Liu, F. L. & Liou, J. G. Zircon as the best mineral for P–T–time history of UHP metamorphism: A review on mineral inclusions and U–Pb SHRIMP ages of zircons from the Dabie–Sulu UHP rocks. *J. Asian Earth Sci.* **40**, 1–39 (2011).

17. Zhang, R. Y., Hirajima, T., Banno, S., Cong, B. L. & Liou, J. G. Petrology of ultrahigh-pressure rocks from the southern Su-Lu region, eastern China. *J. Metamorph. Geol.* **13**, 659–675 (1995).

18. Zheng, Y. F., Fu, B., Gong, B. & Li, L. Stable isotope geochemistry of ultrahigh pressure metamorphic rocks from the Dabie–Sulu orogen in China: Implications for geodynamics and fluid regime. *Earth-Sci. Rev.* **62**, 105–161 (2003).

19. Bell, D. R., Ihinger, P. D. & Rossman, G. R. Quantitative analysis of hydroxyl in garnet and pyroxene. *Am. Mineral.* **80**, 465–474 (1995).
